# Masse géante de l’ovaire en périménopause: à propos d’un cas

**DOI:** 10.11604/pamj.2021.38.232.28240

**Published:** 2021-03-02

**Authors:** Hamza Messaoudi, Khalid Guelzim, Moad Belouad, Abdelhamid Benlghazi, Jaoud Kouach

**Affiliations:** 1 Service de Gynécologie-Obstétrique, Hôpital Militaire d'Instruction Mohammed V, Université Mohammed V Rabat, Rabat, Maroc

**Keywords:** Masse géante, ovaire, périménopause, cystadénome sereux, à propos d’un cas, Huge mass, ovary, perimenopause, serous cystadenoma, case report

## Abstract

Le diagnostic des tumeurs ovariennes géantes survient généralement à un stade avancé surtout dans les pays en voie de développement et peut poser des difficultés opératoire, l´examen anatomopathologique est primordiale pour faire le diagnostic en gros. La valeur du marqueur tumoral Ca125 chez une femme périménopause avec une masse pelvienne a été largement débattue.

## Abstract (en)

Huge ovarian tumor is usually diagnosed at an advanced stage, in particular in developing countries. It can cause surgical complications. Anatomopathological examination is essential to establish the diagnosis. The role of tumor marker CA125 in a perimenopausal woman with pelvic mass has been widely debated.

## Introduction

Les masses ovariennes géantes sont devenues rares en raison d'une découverte fortuite lors des examens radiologiques. Leur prise en charge dépend de la taille de la tumeur, l´âge de la patiente et le type histologique [1]. La fréquence de malignité n'étant que de 37 à 66% chez les femmes periménopausées et de 18 à 86% chez les femmes ménopausées [2,3]. Généralement ces masses géantes donnent des symptômes cliniques avec des signes accompagnateurs, notre cas présente une forme atypique par l´absence des signes cliniques chez une patiente en péri-ménopause.

## Patient et observation

**Figure 1:**
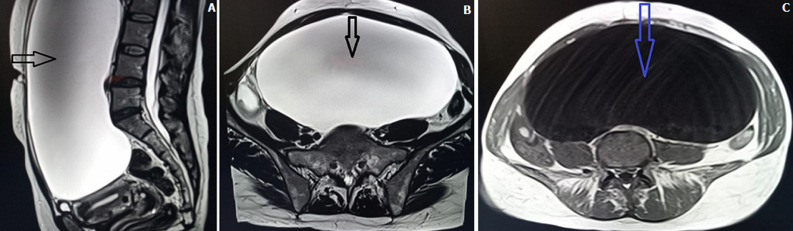
A) coupe transversale en T1, la flèche montrant la volumineuse masse abdomino-pelvienne; B) coupe sagittale en T1, masse abdominopelvienne de nature kystique sans nodulation intrakystique de plus de 19x10cm de grands axes, très bien limité; C) coupe transversale en T2 qui montre une volumineuse masse abdominopelvienne de nature kystique pure non rehaussée après injection

Il s´agissait d´une Patiente âgée de 47 ans, G3P3, ayant trois accouchements par voie basse, avec un cycle qui étais irrégulier sans autre antécédent notable. Elle a été adressée à notre formation pour la prise en charge d´une masse abdominale qui remontait à deux mois sans douleur ni signe urinaire ou digestive associés évoluant dans un contexte d´altération de l´état général et d´amaigrissement chiffré à 6%. L´examen général a objectivé un état hémodynamique stable, apyrétique le poids était à 50 kg, la taille était à 1m60, indice de masse corporelle (IMC): 19, les conjonctives étaient normalement colorées. L´examen abdominale a décelé une distension abdominale sans circulation collatérale, eupnéique, avec une sensibilité à la palpation avec la présence d´une énorme masse abdomino-pelvienne de tonalité liquidienne difficile à limiter. L´inspection vulvo-périnéale était sans particularité et au spéculum révèle un col sain sans saignement ni leucorrhée, le toucher vaginal couplé au palper abdominale a montré la présence d´une énorme masse abdomino-pelvienne mobile et non individualisable par rapport à l´ utérus. Au toucher rectal on perçoit une masse liquidienne prolabée dans le cul de sac du douglas, la cloison recto-vaginale et les paramètres étaient sans particularité. L´échographie pelvienne a objectivé une énorme masse d´allure kystique masquant l´abdomen. Un compliment radiologique par imagerie par résonance magnétique (IRM) pelvienne pour plus de caractérisation. La Figure 1 (A, B, C) qui a montré la présence d´une volumineuse masse abdominopelvienne de nature kystique pure non rehaussée après injection sans nodulation intrakystique de plus de 19x10cm de grands axes, très bien limitée et très compressive, sur les structures adjacentes notamment sur la vessie refoulée en bas et en avant est collabée, et sur l´utérus sans signe d´envahissement évoquant un volumineux kyste ovarien sans signe suspect à son niveau, l’utérus présente une taille normale des contours réguliers.

**Figure 2:**
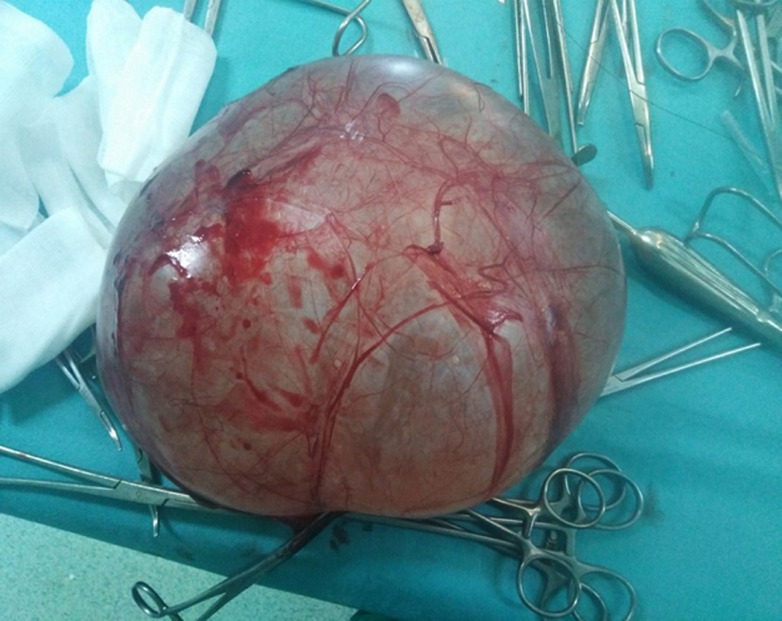
masse kystique encapsulée avec une vascularisation régulière et sa capsule intacte

Sur le plan biologique les marqueurs tumoraux notamment CA125 étaient normaux. Une laparotomie exploratrice a été indiqué devant l´impossibilité de réaliser une coelioscopie vu la taille de la masse, sous anesthésie générale avec une incision médian à l´exploration de la cavité abdominale a montré la présence d´une masse kystique a surface lisse de l´ovaire droit arrivant jusqu´à l´appendice xiphoïde, mobile et non adhérente aux structures adjacentes (Figure 2). L´utérus était de taille normale, l´ovaire gauche, le foie, l’estomac, l’épiploon étaient sans particularité. On a réalisé une cytologie péritonéale et une biopsie épiploïque et péritonéale avec une annexectomie droite. La postopératoire était simple. L´étude anatomopathologique a objectivé sur le plan macroscopique: une masse kystique mesurant 30x23x12 cm à paroi fine et de contenu liquidien (eau de roche) et l´examen microscopique: ses fragments sont composés d´une cavité kystique bordée d´un épithélium cubique surmonté par une bande de tissus conjonctifs fibreux; à certains endroits on observe des bouts de parenchyme ovarien normal en faveur d´un cystadénome séreux et une absence de signe histologique de malignité. Les biopsies épiploïques, péritonéales et cytologie péritonéale étaient indemnes de toute infiltration tumorale.

## Discussion

La particularité de notre observation est la présence d´une énorme masse géante chez une patiente en phase de périménopause avec une évolution lente de la symptomatologie, la fréquence de malignité est de 37 à 66%, et la taille de la tumeur qui oriente vers une pathologie maligne. Les cystadénomes séreux produisent des symptômes non spécifiques. Les symptômes les plus courants comprennent une sensation de pression dans la partie inférieure de l'abdomen et des symptômes du système gastro-intestinal et urinaire. Une douleur aiguë peut survenir en cas de torsion annexiel ou en cas de rupture de kyste [4].

Les tumeurs séreuses se développent par invagination de l'épithélium de surface de l'ovaire et sécrètent du liquide séreux. Généralement bénignes; 5 à 10% ont un potentiel malin limite et 20 à 25% sont malignes [5]. La plupart des patientes arrivent à un stade avancé vu l´intimité des organes génitaux surtout dans les pays en voie de développement, qui rend le diagnostic tardif et peut poser des difficultés opératoire, en plus le siège de l´ovaire au niveau du pelvis pose un problème de diagnostic précoce. Afin de diagnostiquer les tumeurs ovariennes différentes techniques d´imagerie sont utilisées notamment l´échographie pelvienne qui est très performante pour nous orienter vers les signes de malignités. Les cystadénome séreux sont généralement multiloculaires. On a recours à l´IRM pour préciser les limites et les adhérences des organes de voisinage. La mesure du marqueur tumoral CA125 peut être utile [6]. De nombreuses affections bénignes comme les fibromes, la grossesse, l'endométriose et les maladies inflammatoires pelviennes peuvent provoquer des niveaux élevés de CA125 [7]. Ce n´est que l´examen anatomopathologique qui fait la distinction entre les tumeurs séreuses bénignes, borderline et malignes, l´examen anatomopathologique est primordial pour faire le diagnostic ainsi que l´expérience du chirurgien. Un système de notation RMI (indice de risque de malignité) a été développé qui utilise des caractéristiques échographiques avec un état ménopausique et une valeur absolue de CA125 pour tenter de prédire le risque de malignité de la masse ovarienne [3, 7].

## Conclusion

La prise en charge des masses ovariennes géantes dépend des données cliniques, histologiques et radiologiques, généralement ces masses sont découverte à un stade avancé, elles restent toujours fréquentes en périmenopause avec une incidence de malignité de 37 à 66%.
